# Histidine-rich glycoprotein function in hepatocellular carcinoma depends on its N-glycosylation status, and it regulates cell proliferation by inhibiting Erk1/2 phosphorylation

**DOI:** 10.18632/oncotarget.4997

**Published:** 2015-08-12

**Authors:** Qinle Zhang, Kai Jiang, Yan Li, Dongmei Gao, Lu Sun, Shu Zhang, Tianhua Liu, Kun Guo, Yinkun Liu

**Affiliations:** ^1^ Liver Cancer Institute, Zhongshan Hospital, Fudan University, Shanghai 200032, China; ^2^ Cancer Research Center, Institute of Biomedical Science, Fudan University, Shanghai 200032, China

**Keywords:** hepatocellular carcinoma, histidine-rich glycoprotein, cell proliferation, glycosylation

## Abstract

Hepatocellular carcinoma (HCC) is the third most common cause of cancer mortality. Significantly downregulated histidine-rich glycoprotein (HRG) during the dynamic stages (WB, WB7, and WB11) of neoplastic transformation of WB F344 hepatic oval-like cells was screened out by iTRAQ labeling followed by 2DLC-ESI-MS/MS analysis. HRG expression was significantly lower in HCC tissues. HRG overexpression in Huh7 and MHCC-97H hepatoma cell lines led to decreased cell proliferation, colony-forming ability, and tumor growth, and increased cell apoptosis. HRG could inhibit cell proliferation via the FGF-Erk1/2 signaling pathway by reducing Erk1/2 phosphorylation. On the other hand, the functional expression of HRG was also dependent on the glycosylation status at its N-terminal, especially at the glycosylation site Asn 125. The glycosylation of HRG may play a key competitive role in the interaction between HRG and heparin sulfate for binding bFGF and activating the FGF receptor. These findings provide novel insights into the molecular mechanism of HRG in HCC.

## INTRODUCTION

Hepatocellular carcinoma (HCC) is the main subtype among all primary liver cancers, accounting for 70–85% of the known liver cancers worldwide [1]. Nevertheless, the mechanism of the carcinogenesis of HCC remains unclear. The cellular origin of cancer has been hypothesized to be from stem cells due to maturation arrest [2]. Hepatic oval cells (HOCs) have been described as multipotent progenitor cells that can differentiate into both hepatocytes and biliary epithelial cells [3]. Many studies suggest that bipotential HOCs in the adult liver are more likely to become the target cells of carcinogens [4, 5]. Furthermore, HOCs are activated and proliferate in chronic inflammation and are involved in hepatocarcinogenesis [6]. Some HCCs might originate due to the differentiation arrest of oval cells [7]. Therefore, the WB-F344 cell line is deemed to be an *in vitro* model of HOCs, and it has been widely used in HCC development research [8, 9].

Histidine-rich glycoprotein (HRG) is a 75-kDa glycoprotein synthesized by liver parenchymal cells that circulate in plasma at relatively high concentrations (100–200 μg/ml) [10, 11]. The protein has two cystatin-like domains at the N-terminus: a histidine-rich region (HRR) and a C-terminal domain [11, 12]. HRG is known to bind a variety of ligands, including heme, divalent cations such as Zn^2+^, tropomyosin, plasminogen, plasmin, fibrinogen, thrombospondin, IgG, FcγR, C1q, heparin, and heparan sulfate (HS) [11]. HRG, as an adapter protein, is implicated in regulating many processes such as angiogenesis, coagulation, fibrinolysis, cell chemotaxis, immune response, apoptotic process, cell adhesion, cell migration, cell growth, and cell proliferation [13–17]. Interestingly, HRG has been found to be downregulated in endometrial carcinoma [18] and ovarian cancer [19]. Furthermore, HRG has also been found to be reduced in the serum of AFP-negative HBV-related hepatocellular carcinoma [20]. In this study, we showed that HRG was downregulated in the process of neoplastic transformation of HOCs (WB-F344 cells).

Glycosylation is one of the most prominent protein posttranslational modifications that can regulate protein function. There are 3 N-glycosylation sites identified on HRG protein, namely, Asn 63, Asn 125, and Asn 344 [21, 22]. Asn 63 and Asn 125 are at HRG N-terminal regions, which interact with heparin sulfate (HS) [11, 23, 24]. This interaction with HS plays a role in regulating cell proliferation [25]. However, it remains unknown whether the glycosylation of the HRG N-terminal domain can influence protein function.

In this study, by quantitative proteomic analysis, we identified and validated the differential expression of HRG in neoplastic transformation of WB-F344 cells. Overexpressed HRG inhibited HCC cell proliferation through the FGF-Erk1/2 pathway, and the glycosylated status of N-terminal domains of HRG was essential for its protein function. This finding implied that HRG might be a tumor suppressor, providing a new insight into the relation between HRG and HCC.

## RESULTS

### Identification of dynamically differentially expressed protein during WB F344 neoplastic transformation

Characteristics of transformed cells are shown in supplementary Fig. 1. Three groups of samples (WB, WB7, and WB11) were labeled with iTRAQ tags, mixed, separated by a strong cation exchange (SCX) column, and analyzed using the RPLC column and QSTAR XL LC/MS/MS system for protein identification and quantification. Eighty-seven proteins were identified by iTRAQ labeling followed by 2DLC-MS/MS analysis (Supplementary Table 1).

### Validation of downregulated HRG in a transformed model and HCC tissues

**Figure 1:**
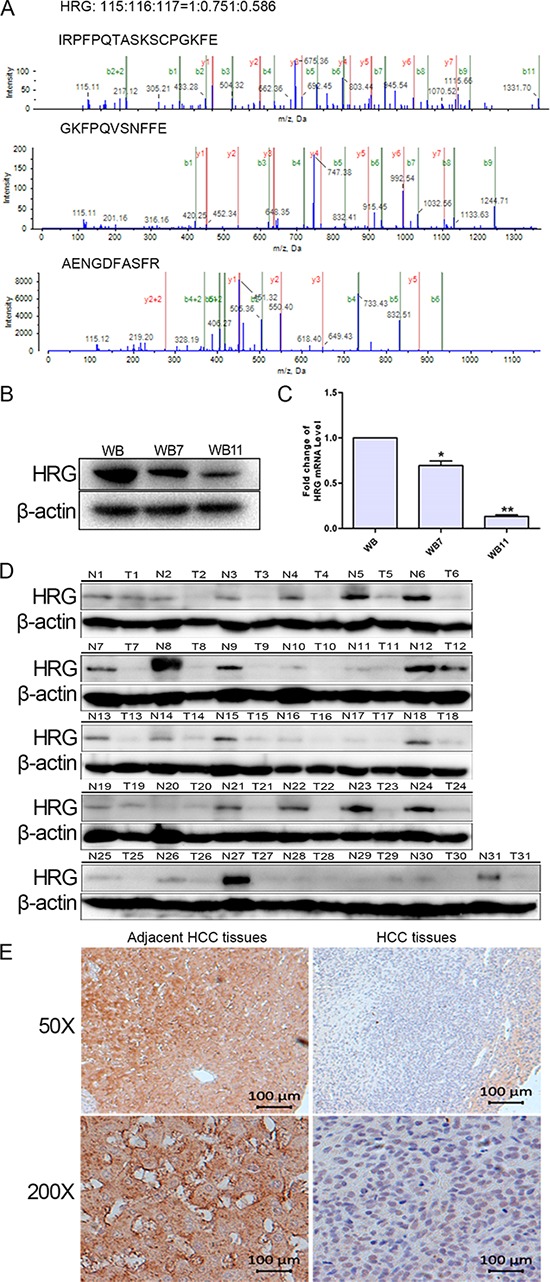
Validation of HRG expression in both neoplastic transformation and HCC tissues **A.** MS/MS analysis of HRG-matched peptides. **B, C.** Western blot analysis and qPCR detected HRG expression in WB, WB7, and WB11. **D.** HRG expression in 31 paired HCC tissues examined by western blotting. **E.** HRG expression in HCC tissue array examined by immunohistochemistry. *p < 0.05, **p < 0.01.

HRG was chosen for further study. We identified 3 matched peptide sequences of HRG as indicated in Fig. 1A. The relative level of HRG was significantly and dynamically downregulated during the transformation processes (WB:WB7:WB11 = 1:0.751:0.586). First, we validated that HRG was downregulated in a transformation model by western blot analysis and quantitative real-time PCR (qPCR) (Fig. 1B and 1C), as well as in 31 paired human HCC tissues and tumor surrounding liver tissues by western blotting. The data showed that HRG was downregulated in 28/31 (90.32%) of the HCC tissue samples (Fig. 1D). Furthermore, we evaluated the HRG levels in additional 75 paired HCC tissues by immunohistochemical staining. The data also showed that HRG was significantly decreased in 70 of 75 (93.33%) HCC tissue samples compared with that of the corresponding adjacent healthy liver tissues (Fig. 1E). These data indicated that HRG was frequently downregulated in HCC samples.

### Overexpressed HRG inhibits cell proliferation, colony formation, and tumor growth, and promotes cell apoptosis in cultured Huh7 and MHCC-97H liver cancer cell lines

**Figure 2:**
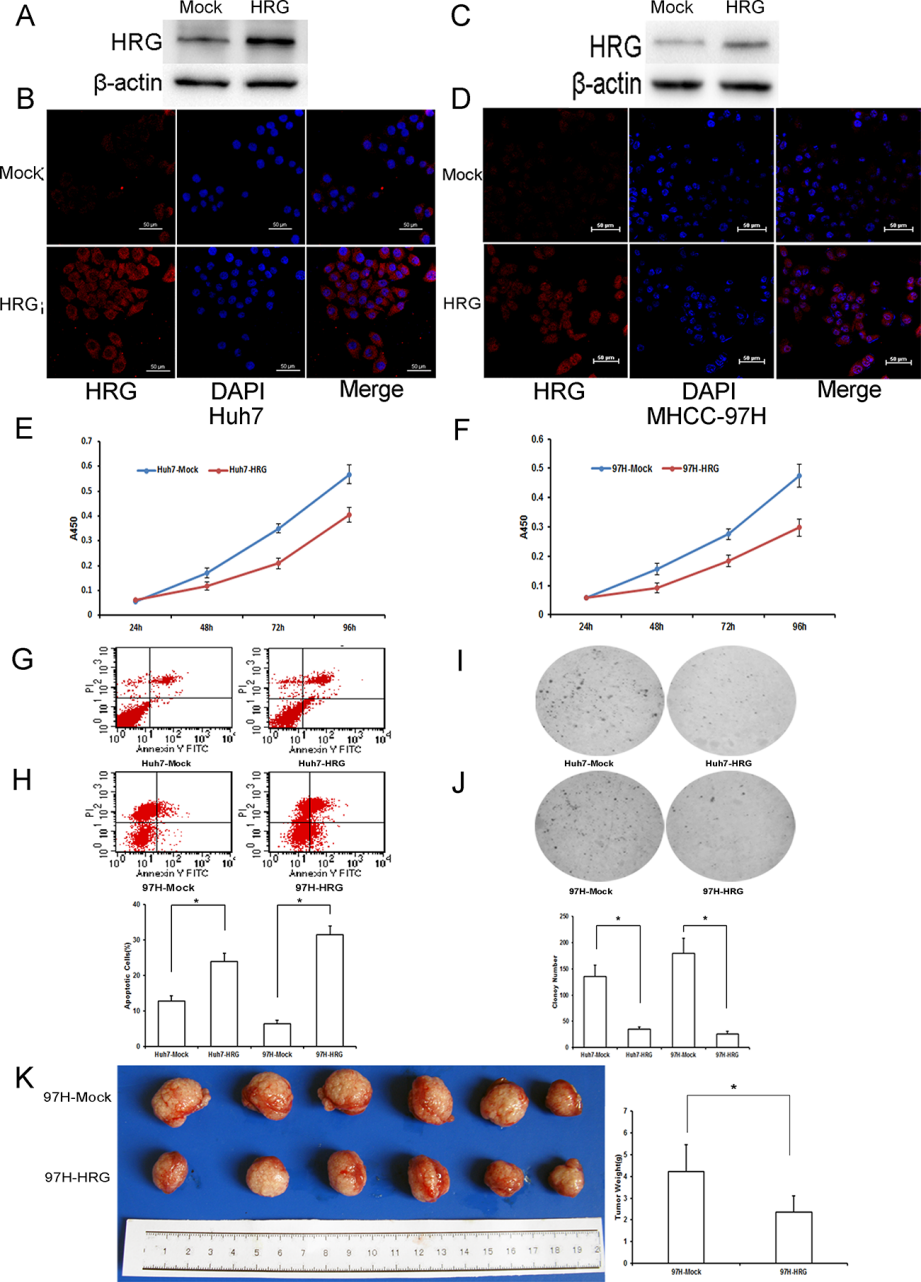
The functional importance of HRG in Huh7 and MHCC-97H hepatocellular carcinoma cells **A–D.** Western blot analysis and immunofluorescence staining were used to detect HRG expression in HRG-transfected Huh7 and MHCC-97H cell lines. **E, F.** On the basis of CCK8 analysis, HRG overexpression in Huh7 and MHCC-97H could inhibit cell proliferation (data are shown as averages ± SD, *n* = 5). **G, H.** Flow cytometry analysis showed that HRG overexpression in Huh7 and MHCC-97H could promote cell apoptosis (data are shown as averages ± SD, *n* = 3). **I, J.** Overexpression of HRG in Huh7 and MHCC-97H could suppress cell soft agar colony formation (data are shown as averages ± SD, *n* = 3). **K.** Overexpression of HRG in MHCC-97H could suppress tumor growth in nude mice. *p < 0.05.

To assess the role of HRG in HCC, we established HRG-transfected Huh7 and MHCC-97H cells by a lentiviral system with puromycin selection. After puromycin selection, HRG expression in Huh7 and MHCC-97H cells was measured by western blot analysis and immunofluorescence microscopy (Fig. 2A–2D). HRG overexpression inhibited the cell viability of Huh7 and MHCC-97H as compared to that of the empty vector control (Fig. 2E and 2F), and on the contrary, promoted cell apoptosis (Fig. 2G and 2H). We performed an anchorage-independent colony formation assay in soft agar to evaluate the effect of HRG on malignant behavior. The result showed that overexpressed HRG significantly inhibited the anchorage-independent growth of Huh7 and MHCC-97H cells in soft agar (Fig. 2I and 2J). We also examined the effect of HRG on tumorigenicity *in vivo* using an orthotopic liver tumor model in nude mice. Compared to the control group, HRG overexpression resulted in significant inhibition of tumor growth (Fig. 2K).

### Inhibitory effect of HRG on FGF-Erk1/2 signaling by the suppression of Erk1/2 phosphorylation

**Figure 3:**
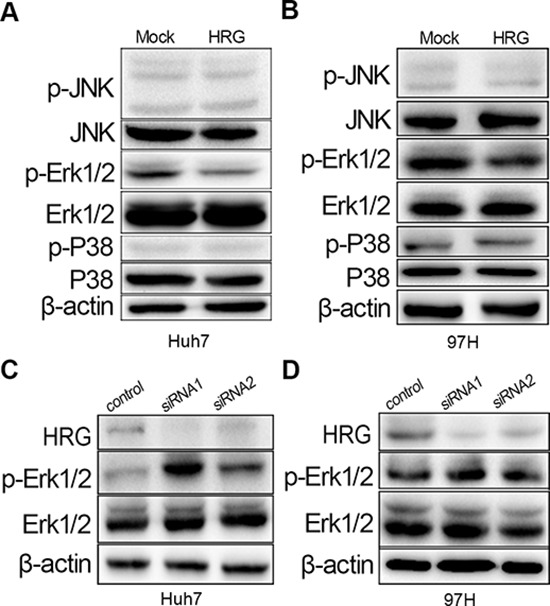
HRG could inhibit cell proliferation via the MAPK-Erk1/2 signaling pathway by reducing Erk1/2 phosphorylation **A, B.** Overexpression of HRG in Huh7 and MHCC-97H could suppress Erk1/2 phosphorylation with no effect on JNK/P38 phosphorylation. **C, D.** Erk1/2 phosphorylation increased in HRG-depleted Huh7 and MHCC-97H cell lines.

To determine the molecular mechanism of how HRG inhibits proliferation, we focused on the FGF-MAPK signaling pathway, which is known to play a major role in HCC cell proliferation [26]. Overexpressed HRG could reduce the phosphorylation of Erk1/2 but not of P38 and JNK (Fig. 3A and 3B). Meanwhile, Erk1/2 phosphorylation could be elevated by silencing HRG expression with siRNA (Fig. 3C and 3D).

**Figure 4:**
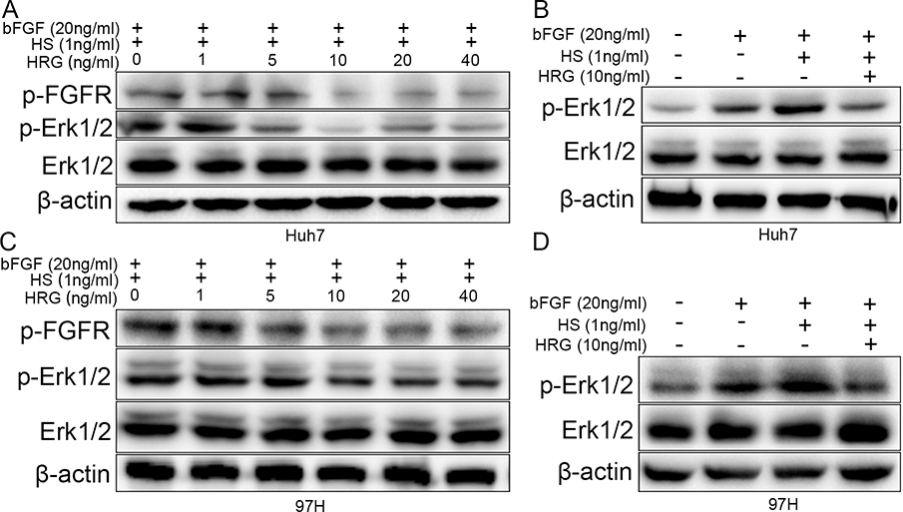
HRG could compete with FGF to bind to HS and finally suppress Erk1/2 phosphorylation via the FGF-Erk1/2 signaling pathway **A, C.** Dose effect of HRG on FGFR and Erk1/2 phosphorylation: 10 ng/ml HRG had the optimum inhibitory effect. **B, D.** The effect of bFGF (20 ng/ml), HS (1 ng/ml), and HRG (10 ng/ml) on Erk1/2 phosphorylation.

FGF first binds to HS and later to FGFR, thereby activating the FGF pathway [27]. Furthermore, HS is a ligand of HRG [11]. We added recombinant proteins bFGF, HS, and HRG to Huh7 and MHCC-97H cells. The result indicated that bFGF and the combination of bFGF and HS could stimulate Erk1/2 phosphorylation, while, most importantly, Erk1/2 phosphorylation returned to a comparatively lower level when HRG was also added (Fig. 4B and 4D). The decrease in Erk1/2 phosphorylation has a dose-dependent relation with HRG, and FGFR phosphorylation also has the similar tendency (Fig. 4A and 4C). These data indicated that HRG could competitively bind to HS and inhibit Erk1/2 phosphorylation via the FGF-Erk1/2 signaling pathway.

### Effect of HRG glycosylation on Erk1/2 signaling

**Figure 5:**
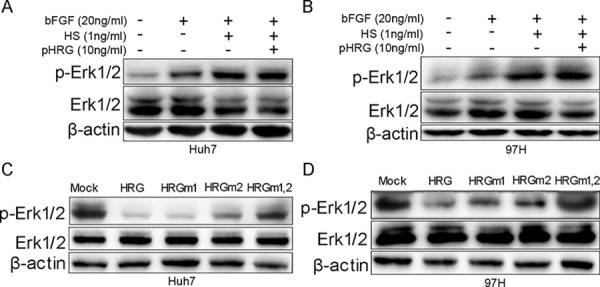
Effect of HRG glycosylation on FGF-Erk1/2 signaling **A, B.** Prokaryotic-expressed recombinant HRG had no effect on Erk1/2 phosphorylation. **C, D.** HRGm1 could inhibit Erk1/2 phosphorylation, as HRG did in Huh7 and MHCC-97H cell lines. HRGm2 had a weaker ability to inhibit Erk1/2 phosphorylation compared to HRG.

To illustrate the role of glycosylation of HRG in its protein function, we added prokaryotic-expressed recombinant HRG, bFGF, and HS to Huh7 and MHCC-97H cells. The HRG without glycosylation could not inhibit Erk1/2 phosphorylation (Fig. 5A and 5B). A series of previous research has shown that HRG N-terminal regions at Asn 63 and Asn 125 glycosylation sites interact with HS [11, 23, 24]. We mutated HRG Asn 63 to Gln63 (termed as HRGm1), Asn 125 to Gln125 (HRGm2), and both sites (HRGm1,2). HRGm1, HRGm2, or HRGm1,2 were then transfected into Huh7 and MHCC-97H cells by a lentivirus system. The result showed that overexpressing HRGm1 could still inhibit Erk1/2 phosphorylation as wild-type (WT) HRG, while partial inhibition by HRGm2 and no inhibition effect by HRGm1,2 (Fig. 5C and 5D). These data suggested that the glycosylation modification on Asn125 in the HRG N-terminal domain played an essential role in interacting with HS and then regulating FGF-Erk1/2 signaling.

### Effect of HRG glycosylation on cell proliferation, cell apoptosis, and colony formation

**Figure 6:**
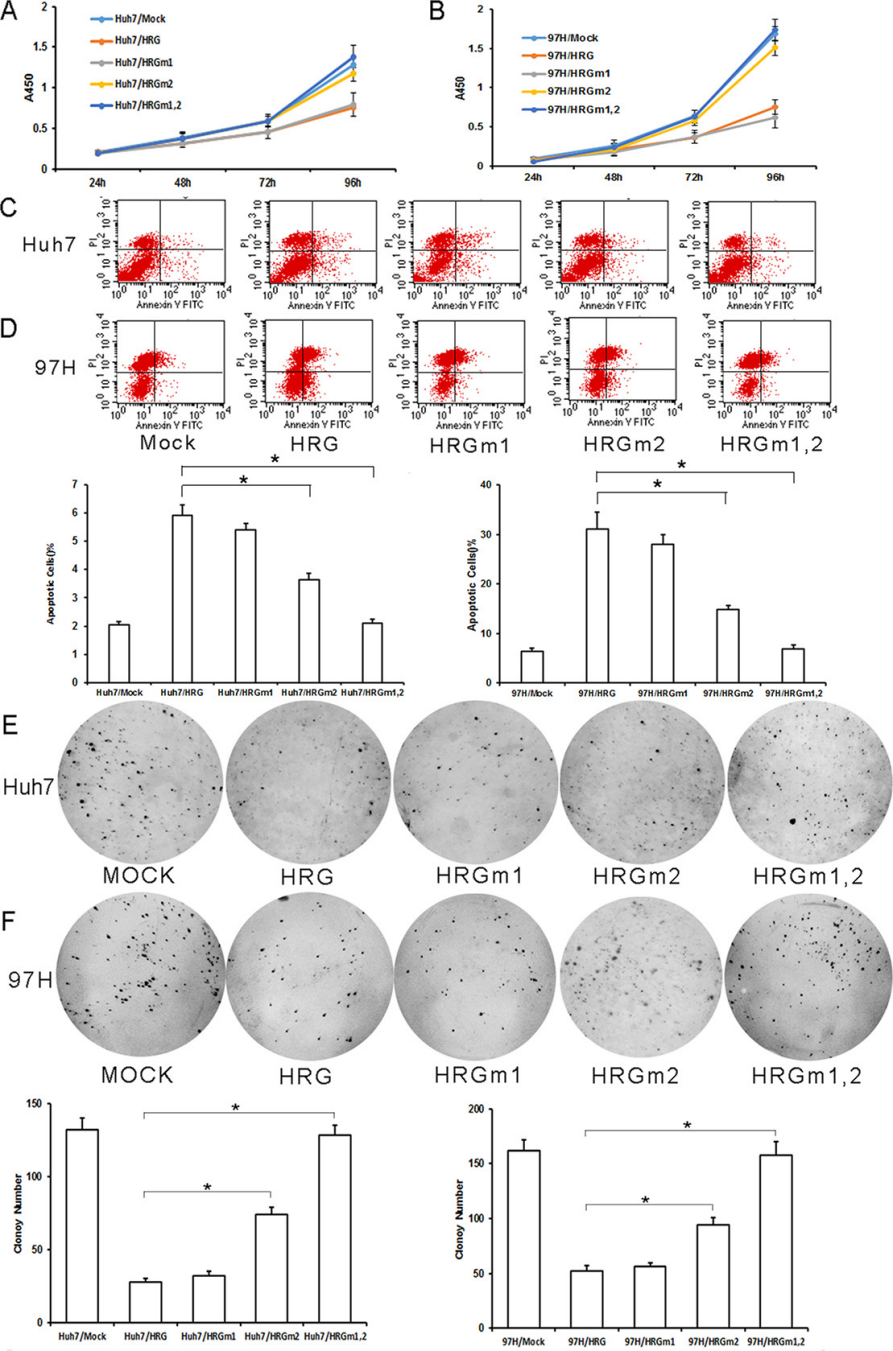
Effect of HRG glycosylation site mutation in Huh7 and MHCC-97H HCC cells **A, B.** According to CCK8 analysis, HRGm1 could inhibit cell proliferation as much as HRG in Huh7 and MHCC-97H cell lines, while HRGm2 could not (data are shown as averages ± SD, *n* = 5). **C, D.** According to flow cytometry analysis, HRGm1 could promote cell apoptosis as much as HRG in Huh7 and MHCC-97H cell lines, while HRGm2 could not (data are shown as averages ± SD, *n* = 3). **E, F.** HRGm1 could inhibit soft agar colony formation as much as HRG, but HRGm2 could not (Data are shown as averages ± SD, *n* = 3). *p < 0.05.

To evaluate the HRG glycosylation in HCC, we overexpressed WT and mutated HRG in Huh7 and MHCC-97H cells. The effect of HRGm1 was similar to that of WT HRG, suppressing cell proliferation and colony formation, and promoting cell apoptosis (Fig. 6A–6F). HRGm2 had a weaker effect in terms of inhibiting cell proliferation, colony formation, and promoting cell apoptosis (Fig. 6A–6F). HRGm1,2 could not inhibit cell proliferation or colony formation and did not promote cell apoptosis at all (Fig. 6A–6F).

## DISCUSSSION

Proteomic profiling was compared between normal WB, pre-cancer status WB7, and early-cancer WB11. All the identified 87 differential expressed proteins were related to cell developmental disorder, organismal injury, immunological disease, hereditary disorder, or cancer. HRG is an abundant plasma protein with functional diversity. Many of these functions are involved in tumor progression and antitumor response. Recent research has found HRG to be at low levels in various types of cancers, such as endometrial carcinoma [18], ovarian cancer [19], and hepatocellular carcinoma [20]. HRG treatment can increase apoptosis and reduce proliferation in fibrosarcoma and suppress endothelial cell migration [28]. Elevated tumor-growing ability of fibrosarcoma and pancreatic carcinomas was observed in HRG-deficient mice than normal mice [29, 30]. In our research, HRG decreased continuously in the process of neoplastic transformation of HOCs, and this trend was validated in HCC tissues by western blot analysis and immunohistochemistry. We performed a series of experiments to evaluate the role of HRG in HCC. The results showed that HRG overexpression in Huh7 and MHCC-97H cells induced marked reductions in cell proliferation, colony formation, and tumor growth, and promoted cell apoptosis. These results suggested that HRG might be an antitumor factor in HCC cells.

HRG can bind multiple ligands such as tropomyosin, plasminogen, plasmin, fibrinogen, thrombospondin, IgG, FcγR, C1q, heparin, and HS [11], thereby modulating immunity, cell adhesion, angiogenesis, thrombosis, apoptotic process, cell adhesion, cell migration, cell growth, and cell proliferation [31]. HCC cell proliferation was mediated by the FGF-MAPK pathway [26, 32]. FGF protein domains include FGF receptor (FGFR)-binding domains and HS-binding domains, which contribute to receptor dimerization [26]. Almost all FGFs require HS as a cofactor to activate FGFR [33, 34]. We hypothesized that HRG might play a role in the FGF-MAPK pathway. Our experimental results were consistent with the hypothesis that HRG overexpression could suppress Erk1/2 activation and HRG silencing could promote Erk1/2 activation. Moreover, HRG competitively bound to the ligand HS rather than bFGF, leading to a decrease in Erk1/2 phosphorylation and FGFR phosphorylation. HRG has been shown to compete with both acidic and basic bFGF for binding to BALBk 3T3 cell-surface HS proteoglycans, and to significantly inhibit FGF-stimulated DNA synthesis [25]. This finding suggests an important role of HRG in inhibiting the FGF-Erk1/2 pathway in HCC cell proliferation.

Glycosylation is one of the most common protein posttranslational modifications. Glycosylation modulates interactions of receptors and ligands, coregulatory molecules, and distinct membrane domains of intact cells, thereby altering signal transduction [35]. As a glycoprotein, HRG has two N-glycosylation modification sites in the N-terminal domain (Asn 63 and Asn 125) [21, 22], and HRG binds to HS via its N-terminal domain [24]. However, the function of the N-glycosylated modification of HRG is not understood. We therefore explored the N-glycosylation function of HRG by N-glycosylation site mutation and found that glycosylation at the N-terminal might play a key role in the interaction between HRG and HS. Mutations of HRG glycosylation sites could affect the activation of Erk1/2, and HRG could not inhibit Erk1/2 phosphorylation without glycosylation, thereby affecting the biological functions of HRG in cell proliferation, cell apoptosis, and colony formation.

In summary, HRG was found to be significantly decreased in HCC during neoplastic transformation of WB-F344 cells. We demonstrated that HRG was probably a negative regulator of HCC carcinogenesis, and HRG regulated cell proliferation via the Erk1/2 signaling pathway. Our data also indicated that glycosylation of HRG played a key role in the interaction between HRG and HS. These findings provided novel insights into the molecular mechanism of HRG in the regulation of HCC.

## MATERIALS AND METHODS

### Cell lines

HCC cell line Huh7 was purchased from the Chinese Academy of Sciences, Shanghai, China. HCC cell line MHCC-97H was established at the Liver Cancer Institute, Zhongshan Hospital, Fudan University. Diploid WB-F344 cells (abbreviated to WB cells) are a rat non-tumorigenic epithelial cell line, gifted generously by Dr. W.B. Coleman (University of North Carolina, Chapel Hill, NC, USA). The cells were maintained in DMEM supplemented with 10% fetal calf serum. The cells were incubated at 37°C in a humidified chamber containing 5% CO_2_.

### Neoplastic transformation of WB-F344 cells

The method of transformation was based on a protocol described previously [9]. Briefly, cell populations were exposed to N-methyl-N′-nitro-N-nitrosoguanidine (MNNG; Tokyo Kasei Kogyo, Tokyo, Japan) during logarithmic growth for 24 h at a final concentration of 5 μg/ml culture medium. Following each exposure, treated populations were allowed to recover from MNNG-induced toxicity and to reach confluence, following which they were split as noted above. One dish was re-treated when it entered logarithmic growth, and the other dishes were used to generate populations for immediate phenotypic analysis and for freezing for future studies. Cells exposed to MNNG 7 or 11 times generated cells called WB7 (pre-cancer stage) and WB 11 (early cancer), respectively.

### Sample preparation, iTRAQ labeling, LC–MS/MS analysis, and immunohistochemistry

The methods for sample preparation, iTRAQ labeling, LC–MS/MS analysis, and immunohistochemical staining are provided in the supplementary materials and methods.

### Construction of HRG lentiviral vectors and glycosylation site mutation vectors

To investigate the effect of HRG overexpression on HCC cell lines, HRG (NM_000412.3) lentiviral vector with a puromycin selection marker was constructed (Genecopoeia Co. Ltd., Guangzhou, China), and empty vectors were used as controls. HRG glycosylation site (Asn 63 and Asn 125) mutation lentiviral vectors were constructed by the Fast Mutagenesis System (TransGen Biotech, Beijing, China). More detail is provided in the supplementary materials and methods.

### RNA interference

Three pairs of siRNAs against HRG were designed. Sequences of siRNA is listed in Supporting Table 1. More detail is provided in the supplementary materials and methods.

### Colony formation

Puromycin (Sigma) was employed to select the transfected HCC cells. More detail is provided in the supplementary materials and methods.

### Western blot analysis, immunofluorescence microscopy, and quantitative PCR

See supplementary materials and methods for details. The primary antibodies and the primers used in this study is provided in Supporting Table 2 and Supporting Table 3.

### Cell proliferation assays and tumor formation assays

See supplementary materials and methods for details.

### Statistical analysis

Statistical analysis was performed with SPSS 15.0 for Windows (SPSS, Chicago, IL, USA). Quantitative variables were analyzed by Student's *t*-test, and *P* < 0.05 was considered statistically significant.

## SUPPLEMENTARY MATERIALS AND METHODS FIGURE AND TABLES


