# Supplementary material for: Histidine-rich glycoprotein function in hepatocellular carcinoma depends on its N-glycosylation status, and it regulates cell proliferation by inhibiting Erk1/2 phosphorylation
Source: Oncotarget. 2015 Aug 12;6(30):30222–31. doi: 10.18632/oncotarget.4997 (PMC4745792; doi:10.18632/oncotarget.4997)
Supplement: Supplementary file 1 [file oncotarget-06-30222-s001.pdf]

SUPPLEMENTARY MATERIALS AND METHODS

SAMPLE PREPARATION, ITRAQ LABELING, AND LC-MS/MS ANALYSIS

The total protein were extracted from 3 groups (WB, WB7, WB11), and protein samples were then reduced, alkylated, digested, and labeled with iTRAQ reagents as previously described. Protein labeled with the iTRAQ tags as follows: WB-115 isobaric tag, WB7–116 isobaric tag and WB11–117 isobaric tag. The LC-MS/MS analysis was performed as described previously. iTRAQ-labeled peptides fragmented to produce reporter ions at 115.1, 116.1 and 117.1, and fragment ions of the peptides were simultaneously produced, resulting in sequencing of the labeled peptides and identification of the corresponding proteins. The ratios of the peak areas of the three iTRAQ reporter ions reflected the relative abundances of the peptides and the proteins in the samples.

IMMUNOHISTOCHEMISTRY ANALYSIS ON TISSUE MICROARRAYS

Tissue array containing 75 HCC samples (Shanghai OUTDO Biotech, China), and mouse anti-HRG monoclonal antibody (R&D Systems, Inc. US) were used in this study.

The process of immunohistochemistry was according to the protocol of HRG antibody. Briefly, paraffin sections were first deparaffinized and then hydrated. After microwave antigen retrieval, as required, endogenous peroxidase activity was blocked with incubation of the slides in 3% H_2O_2, and non-specific binding sites were blocked with 10% rabbit serum. After serial incubation with primary antibody (1:400) and secondary antibody, the sections were developed in diaminobenzidine solution under a microscope and counterstained with hematoxylin. Negative control slides omitting the primary antibodies were included in all assays.

SIRNA TRANSFECTION

Three pairs of predesigned siRNA oligonucleotide against HRG were purchased from Biomics. The sequences of siRNA were listed in Supporting Table 1. Three pairs of siRNA for each target were mixed respectively and transfected into Huh7 cells and MHCC-97H cells at the final concentration of 50 nM using Lipofectamine 2000 (Invitrogen) as manufacturer's instruction. The culturing medium was changed to complete medium after 12 h of transfection and the cells were cultured for another 48 h until detection of gene knockdown.

SOFT AGAR COLONIZATION

For clone formation assay, 1ml of sterilized 0.6% low melting point (LMP) agarose (Sigma) in complete medium was added to one well of 6-well plate first. After the medium became solid gel, 1ml of 0.4% LMP agarose in complete medium with 1000 Huh7 cells and MHCC-97H cells was added on top of the base gel. After culturing for 14 days, clone (>50 cells) numbers were assessed microscopically. All experiments were performed in triplicate.

WESTERN BLOT ANALYSIS

Equal amounts of total proteins (20 μg) were separated by 10% SDS-PAGE and transferred onto PVDF membrane using a Bio-Rad SemiDry apparatus. The membrane was blocked by 5% milk or 2% BSA at room temperature for 1 h. Then, the membrane was incubated with specific primary antibody with suitable dilution at 4°C overnight. The used primary antibodies and their dilution information were listed in Supporting Table 2. After 3 times of 10 min washing by TBST, the membrane was further incubated with HRP-conjugated secondary antibodies (Bio-Rad) at room temperature for 1 h, and then washed again by TBST for 3 times of 10 min. ECL prime Western Blotting Detection Reagents (GE) and ChemiDoc XRS+ system (Bio-Rad) were used to visualize the bands on membrane.

IMMUNOFLUORESCENCE MICROSCOPY

For Immunofluorescence staining, cells grown on glass coverslip were fixed in 4% paraformaldehyde and permeabilized using 0.5% Triton X-100. Then cells were incubated with the primary antibody overnight at 4°C. After thorough washing, cells were then incubated with Alexa-Fluor 555 anti-mouse IgG or anti-rabbit IgG (1:100 dilution, Cell Signaling Technology, Danvers, MA). Finally, cells were washed and stained with DAPI. Images were captured using a Leica fluorescence microscope.

QUANTITATIVE RT-PCR

Total RNA was extracted from cultured cells using TRIzol Reagent (Invitrogen) according to the manufacturer's instruction. 2 μg of total RNA was reversed transcribed into cDNA using RevertAid First Strand cDNA Synthesis Kits (Thermo). cDNA were prepared for subsequent quantitative PCR amplification with

SYBR Premix Ex Taq (TAKARA) using IQ5 (Bio-Rad). The experimental Ct (cycle threshold) was calibrated against that of beta-actin control product. The used paired primers for each gene were listed in Supporting Table 3.

CELL PROLIFERATION ASSAYS

Huh7 cells and MHCC-97H cells (1000 cells/well) were dispensed in 100 μL aliquots into a 96-well plate. At the indicated time points, the 2-(4-indophenyl)-3-(4-nitrophenyl)-5-(2,4-disulphophenyl)-2H-tetrazolium monosodium salt (CCK8, Cell Counting kit) was added to the cells for 1 h, and then the plate was read using an enzyme-linked immunosorbent assay plate reader at 450 nm.

TUMOR FORMATION ASSAYS

Male BALB/C nude mice (5–6 week old) were obtained from Shanghai Institute of Materia Medica (Chinese Academy of Sciences, Shanghai, China). The *in vivo* experiments were carried out strictly in accordance with a protocol approved by the Shanghai Medical Experimental Animal Care Committee (Permit Number: 2009–0082). 1×10^7 cells were injected subcutaneously into the upper left flank region of nude mice. When the tumor reached 1 cm in diameter, they were cut into $2\times2\times2$ mm^3 sized pieces, and implanted into livers of nude mice. The mice were sacrificed at the 27th day after tumor implantation.The tumor size and weight were measured.

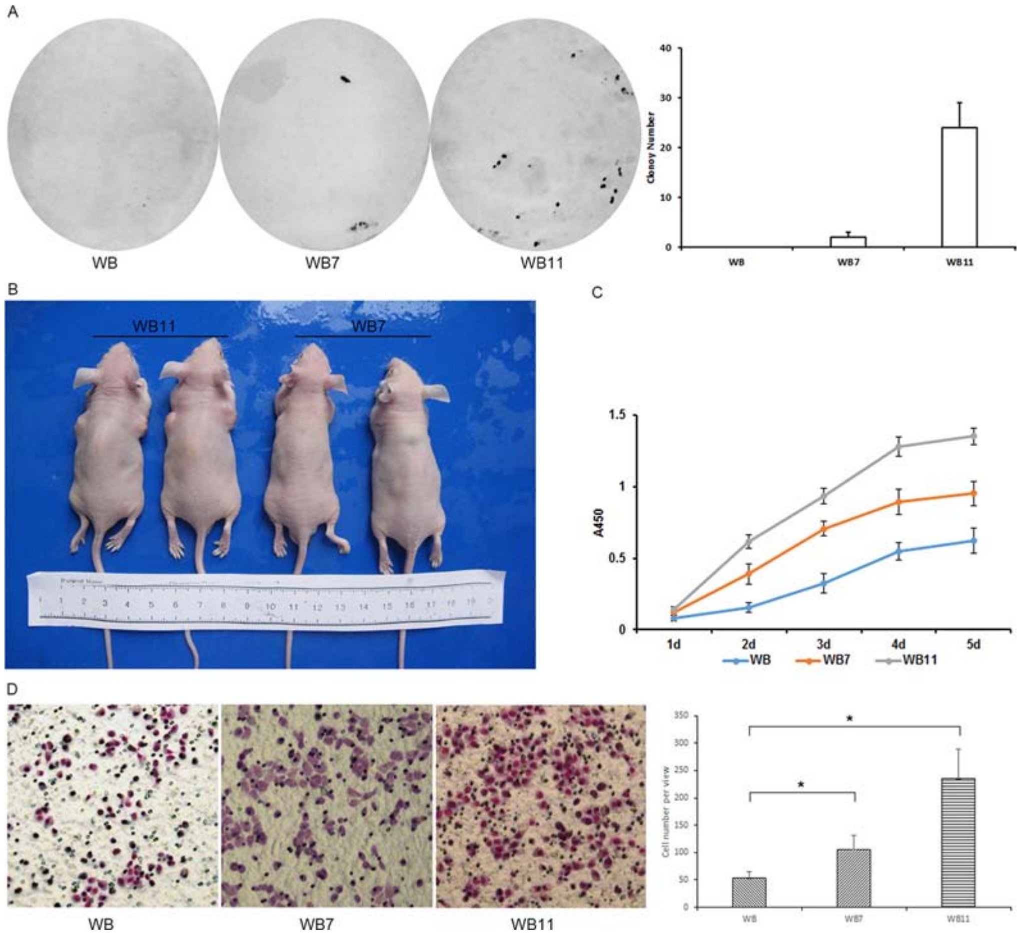

Supplementary Figure S1: Anchoring-independent growth and subcutaneous tumor formation was the important characteristic of transformed cells. The transformed cells could form colonies in soft agar, and the frequencies of colony formation were 0.02% and 2% in WB7 and WB11 cells, but WB cells could not grow in soft agar (Fig. 1A). The subcutaneous tumor formation in WB11 cells, and WB, WB7 cells could not form tumor (Fig. 1B). CCK8 assay displayed that transformed cells proliferated faster than WB cells (Fig. 1C). Cell migration showed that the migration ability was enhanced in WB7 and WB11, and WB11 cells has more migration ability than WB7 cells (Fig. 1D). These results strongly indicated that MNNG induced WB cells gained the characteristics of transformed cells. It implied that the WB7 cell may be in precancer status and WB11 cell, definitely in real cancer. Characteristics of transformed WB, WB7, and WB11 cells. **A.** Soft agar clone formation in WB7 and WB11. **B.** Subcutaneous tumor formation in WB7, WB11. **C.** The proliferation of WB, WB7, WB11 cells. **D.** The migration of WB, WB7, WB11 cells, *$P < 0.05$.

Supplementary Table S1: Sequences of three pair siRNA

Name		Sequences(5′-3′)
SiRNA-1	forward	CGGACAAUGUAAGGUAAUAdTdT
	reverse	UAUUACCUUACAUUGUCCGdTdT
SiRNA-2	forward	CCGGUCCUCAUAGAUUUCUdTdT
	reverse	AGAAAUCUAUGAGGACCGGdTdT
SiRNA-3	forward	GCACCACAAACAUCCUCUAdTdT
	reverse	UAGAGGAUGUUUGUGGUGCdTdT

Supplementary Table S2: List of primary antibodies used in this study

Target protein	Company	Dilution (Application)
HRG	R&D	1:400 (IHC, IF), 1:1000 (WB)
ERK1/2	Cell Signaling	1:1000 (WB)
p-ERK1/2	Cell Signaling	1:1000 (WB),
P38	Sant Cruz	1:100 (WB)
p-P38	Sant Cruz	1:100 (WB)
JNK	Sant Cruz	1:100 (WB)
p-JNK	Sant Cruz	1:100 (WB)
β-actin	Kangchen biotech	1:10000 (WB)
GAPDH	Kangchen biotech	1:10000 (WB)

Supplementary Table S3: List of primers used in realtime RT-PCR (5′ - 3′)

HRG	forward	AGTGTCTTCCAGCCATGAGG
	reverse	TGGTGTTGATATATGCCGAAGAGAC
β-actin	forward	CCCGCGAGTACAACCTTCTT
	reverse	AGGGTCAGGATGCCTCTCTT

Supplementary Table S4: Total 87 proteins were identified from transformation

Uniprot-Accession	Protein Name	Gene Name	115	116	117
P02454	Collagen alpha-1(I) chain	Col1a1	1	1.72	1.33
P02466	Collagen alpha-2(I) chain	Col1a2	1	1.39	1.79
P62630	Elongation factor 1-alpha 1	Eef1a1	1	1.41	1.22
Q99068	Alpha-2-macroglobulin receptor-associated protein	Lrpap1	1	2.17	2.09
P13383	Nucleolin	Ncl	1	1.57	1.47
Q6IFW6	Keratin, type I cytoskeletal 10	Krt10	1	7.73	4.61
P62961	Nuclease-sensitive element-binding protein 1	Ybx1	1	1.24	1.67
Q6IMF3	Keratin, type II cytoskeletal 1	Krt1	1	2.63	1.96
P00762	Anionic trypsin-1	Prss1	1	1.41	1.31
P29457	Serpin H1	Serpinh1	1	1.42	1.21
P13084	Nucleophosmin	Npm1	1	1.24	1.28
Q4KMA2	UV excision repair protein RAD23 homolog B	Rad23b	1	1.56	1.56
Q4FZU2	Keratin, type II cytoskeletal 6A	Krt6a	1	6.79	2.91
Q63945	Protein SET	Set	1	3.40	2.33
P63029	Translationally-controlled tumor protein	Tpt1	1	1.41	2.23
P24368	Peptidyl-prolyl cis-trans isomerase B	Ppib	1	1.85	1.87
P04906	Glutathione S-transferase P	Gstp1	1	2.47	1.50
A0JPM9	Eukaryotic translation initiation factor 3 subunit J	Eif3j	1	1.80	2.40
P06302	Prothymosin alpha	Ptma	1	2.36	2.86
Q6AYK6	Calcyclin-binding protein	Cacybp	1	1.27	1.58
P20059	Hemopexin	Hpx	1	2.83	3.02
Q5RKG1	Spindle and centriole-associated protein 1	Spice1	1	3.40	2.05
Q5I034	Uncharacterized protein C12orf43 homolog		1	1.31	1.56
Q6IUR5	Neudesin	Nenf	1	1.96	1.79
Q80Z30	Protein phosphatase 1E	Ppm1e	1	1.41	1.75
Q4V8I5	ADP-ribosylation factor-like protein 6-interacting protein 4	Arl6ip4	1	1.91	1.51
P35434	ATP synthase subunit delta, mitochondrial	Atp5d	1	3.44	1.82
Q00729	Histone H2B type 1-A	Hist1 h2ba	1	1.25	1.32
Q7TQ84	UAP56-interacting factor	Fyttd1	1	1.33	10.86
P85972	Vinculin	Vcl	1	0.33	0.64
P04937	Fibronectin	Fn1	1	0.70	0.71
P63018	Heat shock cognate 71 kDa protein	Hspa8	1	0.62	0.67
P45592	Cofilin-1	Cfl1	1	0.79	0.68
Q9JI03	Collagen alpha-1(V) chain	Col5a1	1	0.08	0.30
Q9JI85	Nucleobindin-2	Nucb2	1	0.49	0.68

(*Continued*)

Uniprot-Accession	Protein Name	Gene Name	115	116	117
Q07936	Annexin A2	Anxa2	1	0.45	0.46
P05964	Protein S100-A6	S100a6	1	0.58	0.44
Q63610	Tropomyosin alpha-3 chain	Tpm3	1	0.27	0.31
P62260	14–3-3 protein epsilon	Ywhae	1	0.05	0.10
Q9JJ19	Na(+)/H(+) exchange regulatory cofactor NHE-RF1	Slc9a3r1	1	0.38	0.79
P02770	Serum albumin	Alb	1	0.59	0.34
P62982	Ubiquitin-40S ribosomal protein S27a	Rps27a	1	0.75	0.59
P10111	Peptidyl-prolyl cis-trans isomerase A	Ppia	1	0.56	0.22
P07632	Superoxide dismutase [Cu-Zn]	Sod1	1	0.37	0.35
P63259	Actin, cytoplasmic 2	Actg1	1	0.46	0.39
P14668	Annexin A5	Anxa5	1	0.05	0.29
Q8VHK7	Hepatoma-derived growth factor	Hdgf	1	0.25	0.18
P11980	Pyruvate kinase isozymes M1/M2	Pkm	1	0.76	0.66
P09495	Tropomyosin alpha-4 chain	Tpm4	1	0.72	0.37
P05943	Protein S100-A10	S100a10	1	0.47	0.34
P14669	Annexin A3	Anxa3	1	0.76	0.77
P04692	Tropomyosin alpha-1 chain	Tpm1	1	0.52	0.37
Q63081	Protein disulfide-isomerase A6	Pdia6	1	0.59	0.67
O35763	Moesin	Msn	1	0.69	0.48
O08629	Transcription intermediary factor 1-beta	Trim28	1	0.79	0.63
P48679	Prelamin-A/C	Lmna	1	0.29	0.71
P35704	Peroxiredoxin-2	Prdx2	1	0.67	0.76
Q63797	Proteasome activator complex subunit 1	Psme1	1	0.58	0.20
P00507	Aspartate aminotransferase, mitochondrial	Got2	1	0.73	0.79
P61980	Heterogeneous nuclear ribonucleoprotein K	Hnrnpk	1	0.06	0.34
Q64119	Myosin light polypeptide 6	Myl6	1	0.69	0.48
Q499N6	UBX domain-containing protein 1	Ubxn1	1	0.72	0.70
P63100	Calcineurin subunit B type 1	Ppp3r1	1	0.77	0.30
Q9WU49	Calcium-regulated heat stable protein 1	Carhsp1	1	0.68	0.48
P0C0S7	Histone H2A.Z	H2afz	1	0.46	0.56
Q62764	DNA-binding protein A	Csda	1	0.64	0.79
P68511	14–3-3 protein eta	Ywhah	1	0.49	0.47
Q5I0E7	Transmembrane emp24 domain-containing protein 9	Tmed9	1	0.74	0.49
Q6AYH5	Dynactin subunit 2	Dctn2	1	0.41	0.79
P47875	Cysteine and glycine-rich protein 1	Csrp1	1	0.70	0.40

(*Continued*)

Uniprot-Accession	Protein Name	Gene Name	115	116	117
Q63041	Alpha-1-macroglobulin	A1m	1	0.20	0.52
Q63416	Inter-alpha-trypsin inhibitor heavy chain H3	Itih3	1	0.54	0.22
Q99PS8	Histidine-rich glycoprotein	Hrg	1	0.75	0.59
P04785	Protein disulfide-isomerase	P4 hb	1	1.22	0.71
Q6IFU8	Keratin, type I cytoskeletal 17	Krt17	1	3.31	0.77
P31044	Phosphatidylethanolamine-binding protein 1	Pebp1	1	1.41	0.72
P82995	Heat shock protein HSP 90-alpha	Hsp90aa1	1	1.96	0.61
Q9EPH2	MARCKS-related protein	Marcksl1	1	1.34	0.78
P05765	40S ribosomal protein S21	Rps21	1	1.85	0.56
P84039	Ectonucleotide pyrophosphatase/phosphodiesterase family member 5	Enpp5	1	1.66	0.29
P62859	40S ribosomal protein S28	Rps28	1	2.13	0.25
P58775	Tropomyosin beta chain	Tpm2	1	0.63	1.31
P51886	Lumican	Lum	1	0.70	1.46
P02401	60S acidic ribosomal protein P2	Rplp2	1	0.63	1.33
P02600	Myosin light chain 1/3, skeletal muscle isoform	Myl1	1	0.48	2.61
Q920J4	Thioredoxin-like protein 1	Txnl1	1	0.23	1.34
P21263	Nestin	Nes	1	0.32	1.66
